# Supplementary material for: Deep learning motion correction of quantitative stress perfusion cardiovascular magnetic resonance
Source: J Cardiovasc Magn Reson. 2026 Jan 20;28(1):102697. doi: 10.1016/j.jocmr.2026.102697 (PMC13246310; doi:10.1016/j.jocmr.2026.102697)
Supplement: Supplementary file 1 — Supplementary material [file mmc1.docx]

**Supplementary material**

**Imaging parameters**

On the Philips system (a 3-T Achieva TX), imaging was performed with a single-shot spoiled turbo field echo sequence with a sensitivity encoding factor of 2.2 and a reverse-linear profile ordering with a half scan factor of 75%. The field of view was 345 x 345 mm^2^, echo time 1 ms, repetition time 2.2 ms, and flip angle 15°. The acquired resolution was 2.6 x 2.6 mm^2^, reconstructed resolution 1.07 x 1.07 mm^2^, and slice thickness 10 mm. The saturation time was 100 ms for high resolution images and 23.5 ms for low resolution images. The low-resolution AIF slice was acquired with the same acquisition parameters as the high-resolution slices except for the short saturation time

On the Siemens system (a 3T MAGNETOM Vida) imaging was performed with a saturation recovery fast gradient echo sequence using a temporally varying k-space sampling pattern with a total acceleration factor of 5.27 and with a time from saturation to k-space centre 93 ms. The field of view was 380 x 300 mm^2^, echo time 1.17 ms, repetition time 2.62 ms, and flip angle 15°. The acquired resolution was 1.4 x 1.4 mm^2^ and slice thickness 8 mm. The saturation time was 100 ms for high resolution images and 20 ms for low resolution images. The AIF slice used: echo time 0.68 ms, a repetition time 1.18 ms, and flip angle 8°, linear k-space ordering, and a time from saturation to k-space centre 27 ms.

**Data augmentation**

Data augmentations were applied during training of the affine and non-rigid registration models. Hyperparameters of the intensity augmentations were the same during all training and are shown in Supplementary table 1. Gaussian noise with a zero mean and standard deviation of 0.01 is always applied. Besides, the intensity was scaled by a random factor uniformly sampled in the range of -0.3 to 0.3 and shifted by a random offset uniformly sampled in the range -0.2 to 0.2.

*Supplementary table 1: Hyperparameters of intensity augmentations, applied to the first and second affine and non-rigid model of both approaches.*

| Augmentations | Probability | Hyperparameter 1 | Hyperparameter 2 |
| --- | --- | --- | --- |
| Gaussian noise | 1 | Mean: 0 | Standard deviation: 0.01 |
| Scale intensity | 1 | Lower: -0.3 | Upper: 0.3 |
| Shift intensity | 1 | Lower: -0.2 | Upper: 0.2 |

Geometric augmentations were applied during training of the first and second affine registration models. Larger affine augmentations were applied to the first affine model in the pipeline, relative to the affine augmentations applied to the second affine model in the pipeline as can be seen in Supplementary table 2, to encourage the second model to focus on smaller translations and rotations. A random translation range, which is a translation in number of pixels, and a random rotation range, which is an angle in radians, is uniformly sampled from the given ranges for every image during training.

*Supplementary table 2: Hyperparameters of rigid augmentations, different for the first and second affine model.*

| Approach | Probability | Translation range  (nr pixels) | Rotation range  (angle in radians) |
| --- | --- | --- | --- |
| First affine model | 1 | (-20, 20) | (-0.8, 0.8) |
| Second affine model | 0.5 | (-10, 10) | (-0.4, 0.4) |

**Training curves**

**
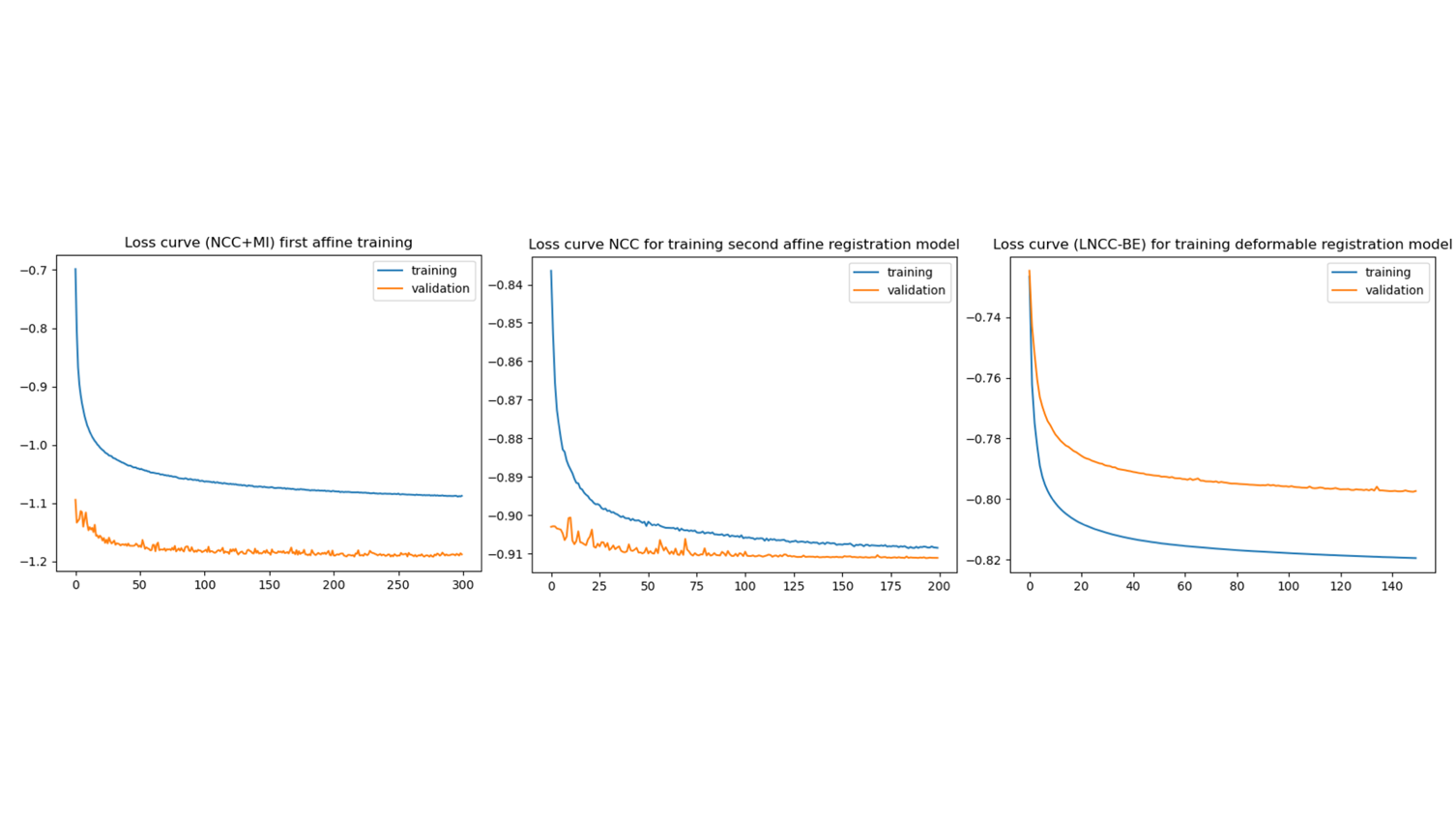
**

***Figure S1:*** Training and validation loss curves over epochs for the proposed registration models. The smooth convergence indicates stable optimization, while the gap between curves in the two affine models reflect that augmentations are used for training but not for validation.

**Jacobian determinant**


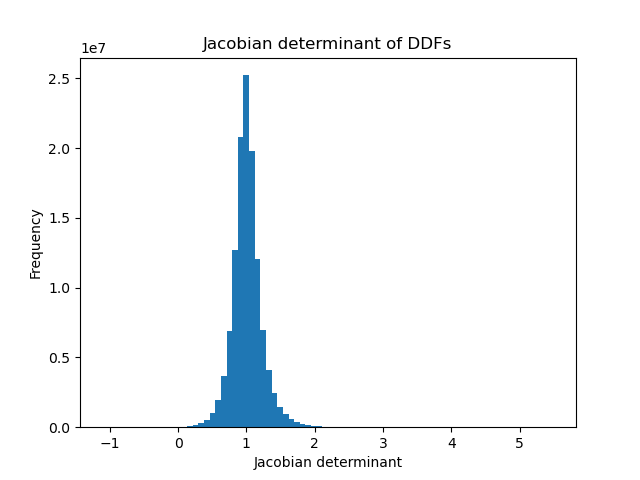


***Figure S2:*** *Distribution of Jacobian determinant values for the predicted deformation fields (DDFs). The mean Jacobian was 1.01 (SD = 0.011), indicating near volume-preserving transformations. Only 0.016% of voxels had negative Jacobian values, suggesting minimal folding and physically plausible deformations.*

**Quantitative perfusion values**

**
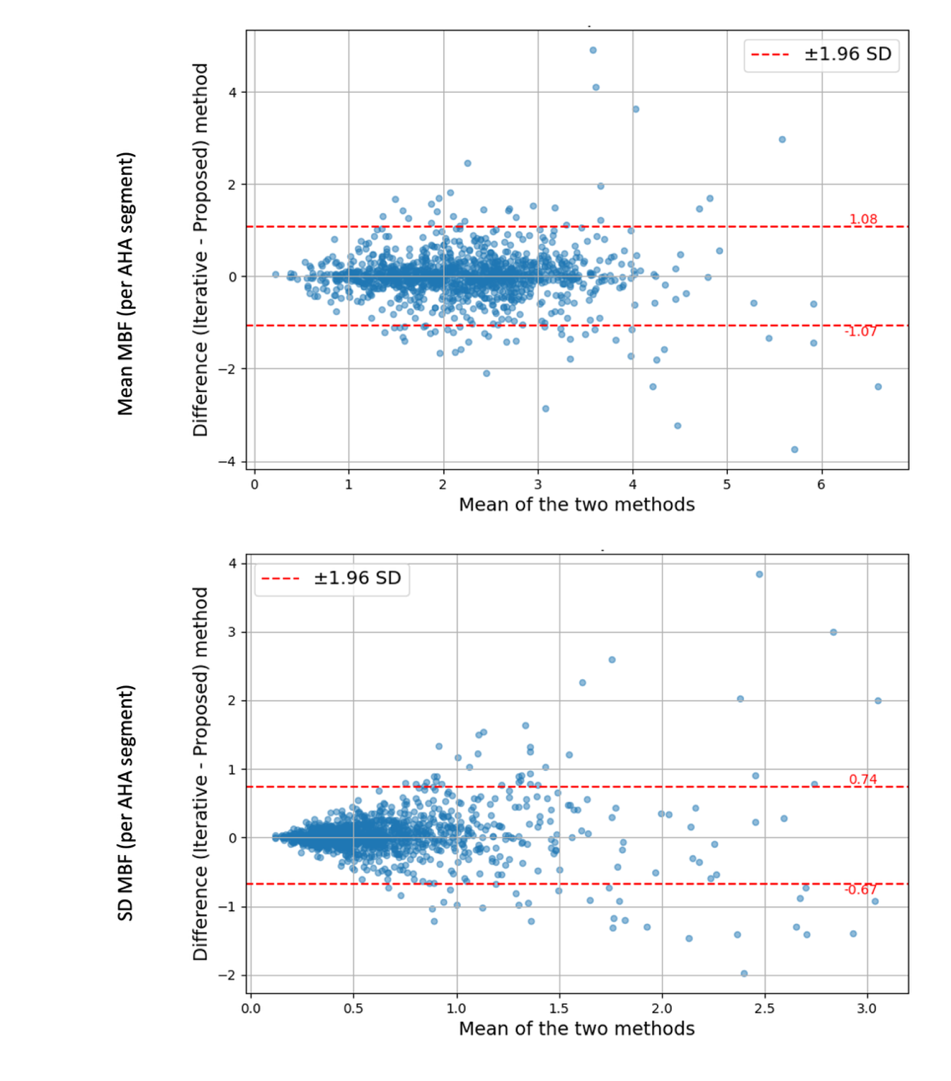
**

***Figure S3:*** *Bland-Altman analysis comparing mean (top) and SD (bottom) quantitative perfusion values computed after iterative registration-based motion correction and the proposed deep learning-based method.*
